# Implementing Anomaly-Based Intrusion Detection for Resource-Constrained Devices in IoMT Networks

**DOI:** 10.3390/s25041216

**Published:** 2025-02-17

**Authors:** Georgios Zachos, Georgios Mantas, Kyriakos Porfyrakis, Jonathan Rodriguez

**Affiliations:** 1 Instituto de Telecomunicações, 3810-193 Aveiro, Portugal; g.mantas@greenwich.ac.uk (G.M.); jonathan@av.it.pt (J.R.); 2 Faculty of Engineering and Science, University of Greenwich, Chatham Maritime ME4 4TB, UK; k.porfyrakis@greenwich.ac.uk; 3 Faculty of Computing, Engineering and Science, University of South Wales, Pontypridd CF37 1DL, UK

**Keywords:** anomaly-based intrusion detection, dataset generation, Internet of Medical Things (IoMT), intrusion detection system (IDS), machine learning algorithms, novelty detection algorithms, outlier detection algorithms

## Abstract

Internet of Medical Things (IoMT) technology has emerged from the introduction of the Internet of Things in the healthcare sector. However, the resource-constrained characteristics and heterogeneity of IoMT networks make these networks susceptible to various types of threats. Thus, it is necessary to develop novel security solutions (e.g., efficient and accurate Anomaly-based Intrusion Detection Systems), considering the inherent limitations of IoMT networks, before these networks reach their full potential in the market. In this paper, we propose an AIDS specifically designed for resource-constrained devices within IoMT networks. The proposed lightweight AIDS leverages novelty detection and outlier detection algorithms instead of conventional classification algorithms to achieve (a) enhanced detection performance against both known and unknown attack patterns and (b) minimal computational costs.

## 1. Introduction

Internet of Medical Things (IoMT) technology has emerged from the introduction of the Internet of Things in the healthcare sector. The purpose of the IoMT is to improve the patient’s quality of life by enabling personalized e-health services without time and location limitations [1,2,3,4]. Nevertheless, the resource-constrained characteristics and heterogeneity of IoMT networks make these networks susceptible to various types of threats, and this, in turn, means that IoMT networks, as well as the healthcare systems relying on these networks, face several security and privacy challenges [5,6]. For example, an attacker may intrude into the IoMT network with the goal of gaining unauthorized access to sensitive information (e.g., medical data). In addition, an adversary may exploit vulnerabilities in the IoMT networks to compromise the corresponding IoT-based healthcare systems in order to disrupt the normal operation of compromised IoT-based healthcare systems (e.g., by flooding the resource-constrained IoMT network with a large number of requests) and/or to tamper with sensing data (e.g., by injecting fake data). This, in turn, can endanger the availability and/or integrity of the healthcare services provided by the compromised IoT-based healthcare systems [2]. Consequently, the development of security solutions protecting IoMT networks from attackers is crucial to ensure the acceptance and wide adoption of IoMT networks in the coming years.

However, conventional security mechanisms are complex and resource-intensive; therefore, they are not suitable for IoMT networks. On the one hand, conventional security mechanisms cannot be afforded by IoMT devices, which are resource-constrained and possess limited processing power, storage capacities, and battery lives. On the other hand, the IoMT devices are deployed and interconnected using lightweight communication protocols in a constrained environment that cannot support conventional security mechanisms [7]. Therefore, to ensure that IoMT networks earn the trust of all stakeholders and realize their full potential in the healthcare sector, it is crucial to create innovative security mechanisms. These mechanisms must effectively and efficiently tackle the significant security challenges faced by IoMT networks, taking into account their unique limitations due to resource constraints [6,8].

To achieve this goal, anomaly-based intrusion detection systems (AIDSs) are recognized by both industry professionals and researchers as a promising security solution that can significantly enhance the protection of IoT networks. However, for these systems to be effective, it is essential to develop innovative lightweight AIDSs tailored for resource-constrained environments [7,9]. Currently, as far as we are aware, only a limited number of AIDSs (i.e., [10,11,12,13,14,15,16]) have been introduced in the literature for protecting resource-limited IoMT devices within IoMT networks. In addition, only two of them (i.e., [13,14]) appear to have been implemented. However, they have not been evaluated during runtime. Moreover, most of the proposed AIDSs for IoMT networks in the literature employ conventional classification machine learning (ML) algorithms (e.g., Naïve Bayes or Random Forest) that are more focused on classifying a new record into one of the classes for which the classification algorithms have been trained [17]. In the case of a record that was not part of the training data, conventional classification ML algorithms cannot make accurate predictions.

Towards this direction, in this work, we propose an AIDS specifically designed for resource-constrained devices within IoMT networks. The proposed AIDS consists of two main components: (a) the monitoring and data acquisition (MDA) component, which is specifically engineered to operate on resource-constrained IoMT devices, and (b) the remote detection engine (RDE) component, which functions on the gateway. The proposed AIDS leverages novelty detection and outlier detection algorithms instead of conventional classification algorithms. The runtime performance of the proposed AIDS was evaluated using an IoMT testbed and dataset produced in our previous works [18,19]. The runtime performance of the implemented AIDS was evaluated using custom scripts that launched both known and unknown (i.e., unknown to the trained ML models) attacks. The runtime performance evaluation results showed that the implemented AIDS can achieve (a) enhanced detection performance against both known and unknown attack patterns and (b) minimal computational costs, with CPU and memory usage for the MDA component remaining below 0.1%.

The rest of this work is organized as follows. Section 2 provides the design of the proposed AIDS for resource-constrained IoMT devices including a detailed description of its various components and internal modules. Afterward, Section 3 describes the novelty detection and outlier detection algorithms employed, the training dataset used, and the runtime performance evaluation of the implemented AIDS. Moreover, Section 4 presents a comparison with state-of-the-art AIDSs. Lastly, Section 5 concludes this paper and provides hints for future work.

## 2. Proposed AIDS

### 2.1. System Architecture

**Figure 1:**
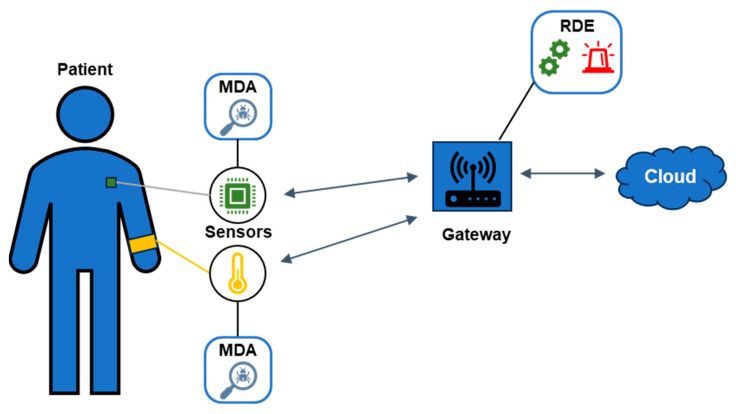
Architecture of the proposed AIDS in the IoMT network.

The proposed AIDS is designed to protect resource-constrained IoMT devices within an IoMT network from both internal and external threats that exploit the inherent security vulnerabilities associated with IoT technology. Our approach not only addresses currently known IoT attack vectors but also potential unknown threats that may emerge in the future, affecting all four layers of the ITU-T IoT reference model [20]. As illustrated in Figure 1, the proposed AIDS consists of two main components: (a) the monitoring and data acquisition (MDA) component, which is specifically engineered to operate on resource-constrained IoMT devices, and (b) the remote detection engine (RDE) component, which functions on the gateway. Detailed descriptions of the MDA and RDE components can be found in Section 2.2 and Section 2.3, respectively.

At this point, it is important to mention that alerts are produced by the RDE component when intrusions that target the IoMT devices connected to the gateway are detected. These produced alerts are sent to a cloud server for further processing and visualization, as depicted in Figure 1. Additionally, the gateway, where the RDE component runs, is required to have enough computational resources (e.g., Raspberry Pi 4 Model B) to support both its standard operations as a relay node and the functions of the RDE component running on it.

In addition, each IoMT device that incorporates the MDA component must meet several essential requirements to function effectively. First, it should have the capability to access its own behavioral data, such as CPU usage and memory utilization. This access is crucial for monitoring performance and identifying potential issues. Second, the device must possess adequate communication bandwidth to efficiently transmit the collected behavioral data to the gateway. This ensures timely and accurate data transfer, which is vital for continuous monitoring and analysis. Lastly, the device needs sufficient computational resources to manage its primary functions as a sensor or actuator while simultaneously supporting the operations of the MDA component. For the implementation of the MDA component, we utilized a Raspberry Pi 4 Model B, which serves as an exemplary IoMT sensor device capable of generating humidity and temperature sensing data. This choice of hardware not only demonstrates the practical application of the MDA component but also highlights its effectiveness in a resource-constrained environment.

### 2.2. Monitoring and Data Acquisition (MDA) Component

The MDA component runs on an IoMT device connected to the gateway. The MDA component performs two operations: (a) monitoring of the behavior of the IoMT device, hosting it, and collection of relevant behavior data (e.g., CPU usage and memory usage) during a specific monitoring period (i.e., the sampling period) and (b) transmission of the collected data to the gateway where the RDE component runs. Then, the RDE component on the gateway can identify whether an attack incident has occurred in the IoMT device hosting the MDA component.

**Figure 2:**
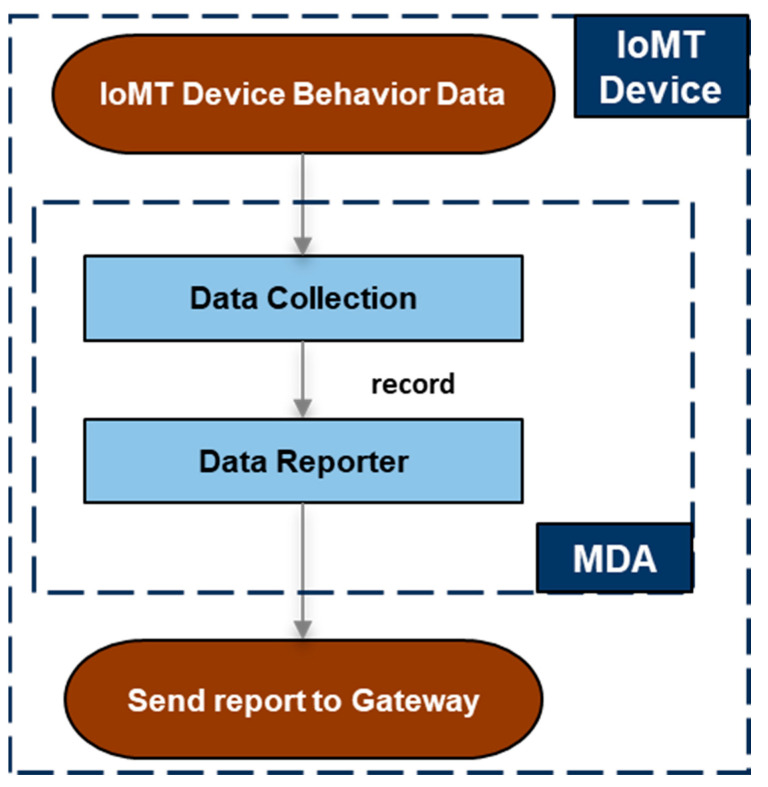
The Monitoring and Data Acquisition (MDA) component.

In essence, the MDA component runs on a resource-constrained IoMT device that cannot perform local intrusion detection on its own. The MDA component was implemented by considering an IoMT device (e.g., Raspberry Pi 4 Model B) running a Linux-based OS, and more specifically, a Debian-based OS (e.g., Ubuntu OS [21]). For the implementation of the MDA component, the C programming language was used to minimize the required resources. In addition, apart from the standard C libraries, the implementation uses one external and freely available library, the Eclipse Paho C client library [22], so that the collected behavior data can be sent to the gateway through the MQTT protocol. The internal architecture of the MDA component, along with its modules, is shown in Figure 2.

#### 2.2.1. Data Collection

The “data collection” module is responsible for the collection of behavior data regarding the IoMT device on runtime during a sampling period (i.e., behavior sampling period). The collected behavior data include the set of features presented in Table 1 along with their descriptions. Based on the collected behavior data, a record in CSV format is created as the output of the “data collection” module.

In particular, the module accesses a specific directory (i.e., “/proc” directory) that is present in a Linux-based OS and gathers the required feature values that are present in the “/proc/stat”, “/proc/meminfo”, and “/proc/diskstats” files. Table 1 presents the feature values collected by the “data collection” module along with their descriptions based on the documentation of the “/proc” directory included in [23,24,25,26].

At this point, it is important to note that some of the collected feature values in Table 1 are related to a specific system resource type (i.e., CPU, memory, or storage) and thus can be organized into the following three feature groups:

- the CPU mode group (i.e., indexes 2–11) containing all feature values related to the time (in ticks) that the CPU spends in a specific mode of operation,
- the memory group (i.e., indexes 17–25) containing all feature values that describe how the system memory is used, and
- the disk stats group (i.e., indexes 26–33) containing all feature values that describe how the OS interacts with storage drives.

#### 2.2.2. Data Reporter

The “data reporter” module receives the collected records from the “data collection” module. For each collected record, a report is created and transmitted to the gateway by the “data reporter” module. The implemented “data reporter” module transmits reports to the gateway using the MQTT application protocol. A report includes two types of information: (i) one line containing a unique identifier of the device where the MDA component is running, and (ii) another line involving the collected record in CSV format. An example of a report is shown below.
**Title:** MDA report (two lines)iomtSensor21733143854599,102899,11863,20796,1155510,1625,0,1016,0,0,0,3532646,11824936,6992,2, 1717639,2035460,124560,610688,614092,886328,826624,126760,8624,20364,68185,5664298, 20705,45733,3789920,75991,0,124190

### 2.3. Remote Detection Engine (RDE) Component

The RDE component runs on the gateway of the IoMT network. The aim of the RDE component is to:

1. receive the MDA reports from the IoMT devices (i.e., hosting the MDA component) that are connected to the gateway and leverage the received MDA reports to identify whether an attack incident has occurred in the connected IoMT devices, and
2. send appropriate security alerts to the cloud server for further processing and visualization when attack incidents are detected.

**Figure 3:**
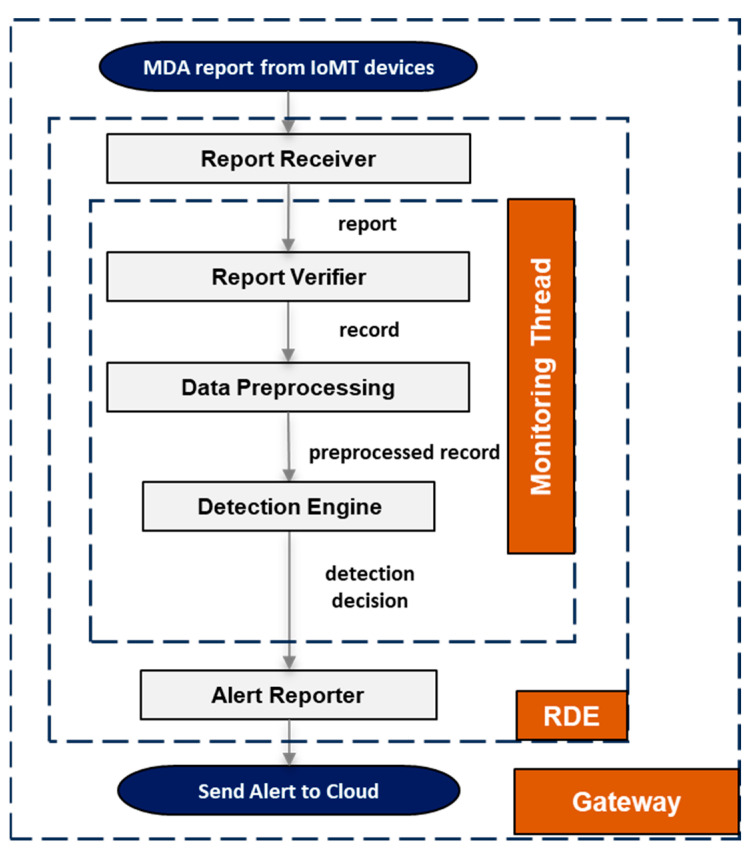
The Remote Detection Engine (RDE) component.

The RDE component was implemented by considering a gateway device (e.g., Raspberry Pi 4 Model B) running a Linux-based OS, and more specifically, a Debian-based OS (e.g., Ubuntu OS [21]). The RDE component is implemented using the Java programming language and the Python programming language. In addition, apart from the standard included Java and Python libraries, the implementation uses the external and freely available libraries described in Table 2. The internal architecture of the RDE component, along with its modules, is shown in Figure 3.

#### 2.3.1. Report Receiver

The “report receiver” module receives an MDA report during runtime on the gateway from a connected IoMT device where the MDA component is running. The purpose of the “report receiver” module is to (a) either create a new CPU thread (i.e., monitoring thread) to process the current MDA report or (b) to redirect the MDA report to an existing processing thread. At this point, it is worth mentioning that the intrusion detection for IoMT devices where the MDA components are running is performed on a per-device basis, meaning that a specific thread created by the “report receiver” module is responsible for performing intrusion detection for only one specific IoMT device based only on the corresponding received MDA reports.

As a new MDA report is received, the “report receiver” module performs the following operations:

1. The “report receiver” module splits the report into its two parts: (a) the unique ID of the IoMT device from where the MDA report originates and (b) the enclosed record collected on the IoMT device.
2. The “report receiver” module checks if the unique ID of the IoMT device is present (i.e., registered) in the configuration file of the RDE component. The configuration file of the RDE component contains an array of unique device IDs that must include all unique IDs of the IoMT devices that are connected to the gateway and where MDA components are running.
3. Only after ensuring that the IoMT device related to the received MDA report is registered in the configuration file of the RDE component does the “report receiver” module proceed to check whether a processing thread regarding this IoMT device already exists.
4. In the case of an existing thread, the new MDA report is redirected to it; otherwise, a new thread is created to process the new MDA report.

#### 2.3.2. Report Verifier

The “report verifier” module processes an MDA report on runtime. The module performs the following operations:

1. The “report verifier” module splits the report into its two parts: (a) the unique ID of the IoMT device from where the MDA report originates and (b) the enclosed record collected on the IoMT device.
2. The “report verifier” module performs a check on the enclosed record to verify whether the record is valid. In particular, the check is performed to ensure that the record follows the CSV format and that it contains the expected number of features with their expected types.
3. If the record is deemed valid, it is forwarded to the “data preprocessing” module. Otherwise, if the record is deemed invalid, it is discarded, and the thread waits for a new MDA report.

#### 2.3.3. Data Preprocessing

The input of the “data preprocessing” module is a record produced by the “report verifier” module. The output of the “data preprocessing” module is a preprocessed record in CSV format that can be used by the “detection engine” module. The “data preprocessing” module processes the input record in two stages to produce the output preprocessed record.

Based on the description of the features in Table 1, it can be observed that some feature values (i.e., features with indexes 1–14, 16, 26–31, and 33) depend heavily on the time that has elapsed since the system boot. As long as the monitored system (i.e., IoMT device) is up and running, these feature values will be continuously increasing. In this context, our focus is not on the actual value of these features but on the increment that has occurred between subsequent sampling periods. Thus, the objective of the first preprocessing stage is the decoupling of the feature values of a collected record from the runtime duration of the underlying system.

The first preprocessing stage processes the values of the features with indexes 1–14, 16, 26–31, and 33. In contrast, the values of the remaining features (i.e., features with indexes 15, 17–25, and 32) do not depend on the runtime duration of the system. Therefore, they are simply forwarded to the second preprocessing stage without being processed in the first preprocessing stage.

Below, we present an appropriate equation to clarify further how the first preprocessing stage works. We assume that the record ($X_{t}^{col}$) collected at time t, containing 33 feature values, is denoted as $X_{t}^{col}\in R_{\geq 0}^{33}$. In addition, we present $X_{t}^{pp1}\in R_{\geq 0}^{33}$ as the record produced after the first preprocessing stage (i.e., pp1) based on $X_{t}^{col}$. In addition, let $x_{t,i}^{col}\in R_{\geq 0}$ denote the $i^{th}$ feature value of $X_{t}^{col}$, where $i\in N^{*}$ is an integer in range [1, 33]. Furthermore, we assume that $x_{t,i}^{pp1}\in R_{\geq 0}$ represents the corresponding output feature value, after the first preprocessing stage, for the feature value $x_{t,i}^{col}$. Finally, we assume that the first record is collected at t=0. Then, Equation (1) shows how $x_{t,i}^{pp1}$ is computed based on $x_{t,i}^{col}$ for different moments in time and different features indexes. Furthermore, the output feature values of a record, after the first preprocessing stage, are summarized in Table 3.

(1) $$x_{t,\;i}^{pp1}=\left\{\begin{array}{c} x_{0,\;i}^{col},\;\;\;t=0\\ x_{t,\;i}^{col},\;\;\;t>0,\;i\;\in \left\{15\right\}\;\cup \;\left\{17,\ldots ,25\right\}\;\cup \;\left\{32\right\}\\ x_{t,\;i}^{col}-x_{t-1,\;i}^{col},\;\;\;t>0,\;i\;\in \left\{1,\ldots ,14\right\}\;\cup \;\left\{16\right\}\;\cup \;\left\{26,\ldots ,31\right\}\;\cup \;\left\{33\right\}\; \end{array}\right.$$

The output of the first preprocessing stage is received by the second preprocessing stage that, in turn, performs normalization on: (a) the feature values of the “CPU mode” feature group, (b) the feature values of the “memory” feature group, and (c) the output “delta values” of the first preprocessing stage based on the “timestamp_delta” value (i.e., the feature value with index 1). The objective of the second preprocessing stage is the decoupling of the feature values of a record produced by the first preprocessing stage from the collection sampling period of the “data collection” module of the MDA component.

Below, we present an appropriate equation to further clarify how the second preprocessing stage works. We denote $X_{t}^{pp1}\in R_{\geq 0}^{33}$ as a record generated by the first preprocessing stage at time t containing 33 feature values. In addition, we define $X_{t}^{pp2}\in R_{\geq 0}^{31}$ as the record produced by the second preprocessing stage (i.e., pp2), containing 31 feature values, based on $X_{t}^{pp1}$. In addition, let $x_{t,i}^{pp1}\in R_{\geq 0}$ represent the $i^{th}$ feature value of $X_{t}^{pp1}$, where $i\in N^{*}$ is an integer in range [1, 33]. Similarly, we consider $x_{t,k}^{pp2}\in R_{\geq 0}$ as the $k^{th}$ feature value of $X_{t}^{pp2}$, where $k\in N^{*}$ is an integer in range [1, 31]. Finally, we consider that the first record is collected at t=0. Then, Equation (2) shows how $x_{t,k}^{pp2}$ is computed based on $x_{t,i}^{pp1}$ for different feature indexes.

(2) $$x_{t,\;k}^{pp2}=\left\{\begin{array}{c} \frac{x_{t,\;i}^{pp1}}{\sum_{m=2}^{11}x_{t,\;m}^{pp1}},\;\;\;t\geq 0,\;k\;\in \left\{1,\ldots ,10\right\},\;i=k+1\;(cpu\;mode\;feature\;group)\\ \frac{x_{t,\;i}^{pp1}}{x_{t,\;1}^{pp1}},\;\;\;t\geq 0,\;k\;\in \left\{11,12,13,15\right\},\;i=k+1\\ x_{t,\;i}^{pp1},\;\;\;t\geq 0,\;k=14,\;i=15\\ \frac{x_{t,\;i}^{pp1}}{x_{t,\;17}^{pp1}},\;\;\;t\geq 0,\;k\;\in \left\{16,\ldots ,23\right\},\;i=k+2\;(memory\;feature\;group)\\ \frac{x_{t,\;i}^{pp1}}{x_{t,\;1}^{pp1}},\;\;\;t\geq 0,\;k\;\in \left\{24,\ldots ,29\right\}\;\cup \left\{31\right\},\;i=k+2\\ x_{t,\;i}^{pp1},\;\;\;t\geq 0,\;k=30,\;i=32 \end{array}\right.$$

At this point, it is worth explaining the above Equation (2) further. In a record produced by the first preprocessing stage, the values of the “CPU mode” feature group (i.e., feature indexes 2 to 11) detail the time (i.e., in ticks) that the CPU has spent in different modes of operation during the last behavior sampling period. During the second preprocessing stage, the feature values of the “CPU mode” group are summed, with the sum being equal to the total CPU ticks during the last behavior sampling period. Then, each feature value of the “CPU mode” group (i.e., feature indexes 2 to 11) is divided by the computed sum. The ten computed feature values are among the output values of the second preprocessing stage, and they describe the percentage of CPU ticks that the CPU has spent in a specific mode of operation during the last behavior sampling period.

Afterward, regarding the values of the “memory” feature group (i.e., feature indexes 17 to 25), almost every value (i.e., feature indexes 18 to 25) is divided by the value of the “memTotal” feature (i.e., feature index 17). The eight computed feature values are among the output values of the second preprocessing stage, and they describe the memory usage as percentages in the range [0, 1].

Moreover, the values of features with indexes of 12–14, 16, 26–31, and 33 from the first preprocessing stage are divided by the value of the elapsed time between two collected records (i.e., the “timestamp_delta” value in index 1). The eleven computed feature values are among the output values of the second preprocessing stage. As a result of the performed division process, the eleven computed values do not depend anymore on the magnitude of the elapsed time between two collected records.

Lastly, the remaining feature values from the first preprocessing stage (i.e., feature values with indexes 15 and 32) are not processed in the second preprocessing stage and thus are simply forwarded to the output.

The output feature values of a record after the second preprocessing stage are the output of the “data preprocessing” module and are summarized in Table 4.

#### 2.3.4. Detection Engine

**Figure 4:**
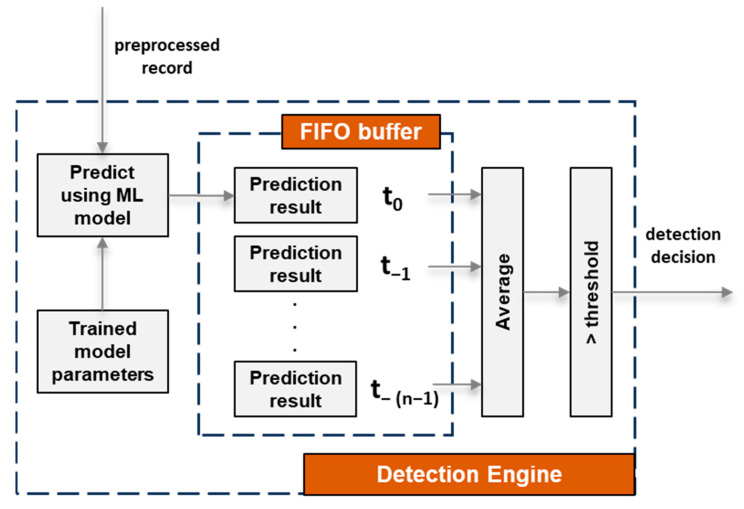
Internal architecture of the “Detection Engine” module of the RDE component.

The output of the “data preprocessing” module is a preprocessed record, and it is received by the “detection engine” module as shown in Figure 3. Based on the input preprocessed record, the “detection engine” module detects whether or not an intrusion has occurred on the connected device hosting the MDA component, meaning that the output of the “detection engine” module is a detection decision. In principle, this is the core module of the proposed RDE component and uses ML algorithms to identify both known and unknown attacks targeting the resource-constrained devices connected to the gateway. The internal architecture of the “detection engine” module is depicted in Figure 4.

As shown in Figure 4, for each received preprocessed record, a trained ML model is used to calculate a prediction result. There are two possible values (i.e., “1” or “0”) for the prediction result, signifying whether an intrusion event has occurred or not based on a specific preprocessed record. Subsequently, the calculated prediction result for each record is stored in a FIFO buffer with a length equal to “n”, which is configurable within the RDE component. The FIFO buffer stores the most recent prediction result, as well as the previous “n − 1” prediction results that were computed based on the last “n − 1” preprocessed records. Afterward, an average value is computed based on all the values of the prediction results inside the buffer. The computed value (i.e., intrusion probability) is then compared to a “threshold” value so that the “detection engine” module can determine whether an intrusion event has occurred or not.

In principle, the purpose of the FIFO buffer is to enable the “detection engine” module to consider not only the last received preprocessed record but also a specific number (i.e., n − 1) of previously received preprocessed records for the task of performing intrusion detection. In addition, the length of the FIFO buffer inside the “detection engine” module is configurable and this, in turn, means that the intrusion detection process can be fine-tuned in one more way than by just training a better ML algorithm.

Moreover, as mentioned, a “threshold” value is compared with the intrusion probability calculated by averaging the prediction results in the FIFO buffer to determine whether an intrusion event has occurred or not. The “threshold” value is also controlled by a respective configuration parameter (i.e., “threshold” parameter) of the RDE component. The purpose of the “threshold” parameter is to provide an extra manner of fine-tuning the intrusion detection process of the “detection engine” module. A higher value for the “threshold” parameter means that positive decisions (i.e., decisions that an intrusion event has occurred) will occur only at a higher intrusion probability value and vice versa.

Lastly, it is important to mention that the “detection engine” module makes a prediction based on one preprocessed record by using a custom Python script. The functionality of the custom Python scripts follows three steps:

1. Parse the input preprocessed record,
2. Load the trained ML model, and
3. Make a prediction.

The use of custom Python scripts increases the ease and flexibility of integrating different ML algorithms into the “detection engine” module of the RDE component.

#### 2.3.5. Alert Reporter

The input of the “alert reporter” module is the detection decisions from the “detection engine” module. When an intrusion is detected, the “alert reporter” module sends an appropriate alert, in JSON format, to the cloud server. The “alert reporter” module is implemented to be able to send alerts to the cloud server through either the HTTP application protocol or the MQTT application protocol. Each alert contains three key pieces of information: (i) a timestamp indicating the time that the intrusion incident occurred, (ii) a unique identifier of the device hosting the MDA component, and (iii) the intrusion probability, represented as a decimal number within the range [0, 1], which reflects the likelihood that an intrusion incident has occurred. An example of an alert, in JSON format, produced by the RDE component for a connected resource-constrained device (i.e., “iomtSensor1”), hosting the MDA component, is shown below.
**Title:** RDE intrusion alert based on detection decisions in JSON format   {    “ts”: “2024-10-04 10:13:34”,    “dev_ID”: “iomtSensor1”,    “intrusion_prob”: “1.0”   }

## 3. Runtime Performance Evaluation

The proposed AIDS was implemented on the IoMT security testbed that was developed as part of our previous works in [18,19]. The next step is to train and integrate novelty detection and outlier detection algorithms into the “detection engine” module of the RDE component in order to evaluate the performance of the implemented AIDS during runtime. The runtime performance evaluation focuses on two key aspects: (a) assessing the performance of the ML models in detecting attacks during runtime and (b) measuring the computational cost of the implemented AIDS components on the IoMT device (i.e., MDA component) and the gateway (i.e., RDE component).

### 3.1. Employed Detection Algorithms, Training Dataset, and Hyperparameters

At this point, it is necessary to discuss why the focus is on novelty detection and outlier detection algorithms in this work. This is because these algorithms need to be trained mostly on records from a single known class to determine whether a new record is similar to the training records [17,31]. In our context, it is feasible to collect training records that represent the normal operation of the IoMT network, which corresponds to a single known class. On the other hand, obtaining training records related to IoMT networks under attack (i.e., known or unknown attacks) is not possible. This limitation underscores the necessity for advanced detection algorithms, such as novelty detection and outlier detection algorithms, capable of identifying anomalies without relying on labeled datasets from attack scenarios. By focusing on these algorithms, we aim to enhance the intrusion detection performance in IoMT networks, where labeled attack data are unavailable.

In addition, the aim is to use algorithms that are already implemented and readily available for use. This leads to focusing on novelty detection and outlier detection algorithms available in two popular Python libraries, namely Scikit-Learn [32,33] and PyOD [34,35]. These libraries provide robust implementations of these algorithms, facilitating the integration into the proposed AIDS.

Furthermore, regarding the novelty detection and outlier detection algorithms that are to be integrated into the RDE component (i.e., the “detection engine” module), we are using a dataset generated in our work in [19], along with the hyperparameters for training these algorithms as presented in [19]. In [19], a dataset (i.e., the LDE dataset) was generated by capturing behavior records from an IoMT device (i.e., the Raspberry Pi device) that was meant to function as a sensor device, similarly to the IoMT sensor device (i.e., the Raspberry Pi device) hosting the MDA component. In addition, the “LDE dataset” contains records with the same features as those used by the “detection engine” module of the RDE component. Thus, in this case, we trained the employed algorithms using the “LDE dataset” and applied the same hyperparameters for these algorithms as investigated in [19].

Based on our work in [19], we trained and evaluated the runtime performance of five novelty detection algorithms and one outlier detection algorithm: (i) one class support vector machine (OCSVM), (ii) local outlier factor (LOF), (iii) Gaussian kernel density estimation (G_KDE), (iv) Parzen window kernel density estimation (PW_KDE), (v) Bayesian Gaussian mixture models (B_GMM), and minimum covariance determinant (MCD). Moreover, based on our work in [19], Table 5 shows the hyperparameters used when training the six detection algorithms.

### 3.2. Runtime Performance Evaluation Methodology

The performance evaluation was performed during various sessions (i.e., evaluation sessions). During each session, a trained ML model is integrated into the RDE component and its performance is evaluated during runtime. More specifically, we followed the steps described below during each evaluation session:

1. It is ensured that all the components of the IoMT testbed are up and running.
2. One detection algorithm is selected from Table 5, along with its hyperparameter set. The “LDE dataset” is used to train a corresponding ML model that is then integrated into the “detection engine” module of the RDE component of the AIDS.
3. The RDE component is executed on the Raspberry Pi 4 device acting as a gateway.
4. The MDA component is executed on the Raspberry Pi 4 device acting as a sensor device.
5. We measure the CPU usage and memory usage of the MDA component on the IoMT device, hosting it by executing a custom bash script (i.e., the “cpu-mem usage” script) on the IoMT device during runtime.
6. We measure the CPU usage and memory usage of the RDE component on the gateway by executing the “cpu-mem usage” script on the gateway during runtime.
7. We launch attacks against the IoMT device hosting the MDA component by executing custom scripts.
8. The successful (or unsuccessful) detection of the launched attacks is verified by checking both the logs generated by the attack scripts during execution as well as the internal logs produced by the AIDS.

At this point, it is worth describing how attacks were launched against the IoMT device hosting the MDA component. During the evaluation, we created artificial resource consumption on the IoMT device of the testbed by executing the stress-ng tool [36]. This artificial resource consumption is designed to replicate a scenario where an adversary executes an attack that exhausts the computational resources of the IoMT device. The documentation of the stress-ng tool presented in [37] is used, and we execute the stress-ng tool on the IoMT device using the following two commands during the evaluation sessions. It is worth mentioning that the following two commands were used to assess the performance of the implemented AIDS against attacks included in the training dataset of the ML model.
stress-ng --cpu 1 --vm 1 --vm-bytes 512Mstress-ng --cpu 2 --vm 2 --vm-bytes 512M

In addition, during evaluation sessions, the following new commands were used to assess the performance of the implemented AIDS against attacks that were not included in the training dataset of the ML model.
stress-ng --matrix 4 --matrix-size 64 stress-ng --vm 2 --vm-bytes 2G --mmap 2 --mmap-bytes 2Gstress-ng --timer 32 --timer-freq 1000000

During the evaluation, each command was executed for a certain period of time (e.g., 4 min) and periods with no command execution were mixed in. The purpose of using two different groups of commands is to measure the performance of the AIDS in detecting both known and unknown attack patterns.

### 3.3. Runtime Performance Evaluation of Detection Algorithms

**Figure 5:**
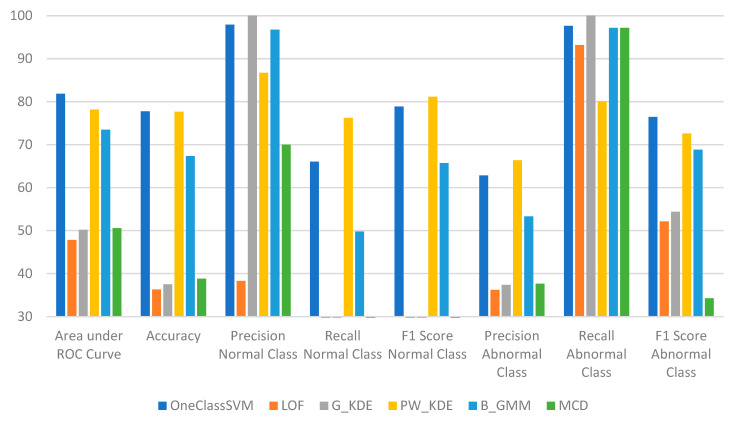
Runtime evaluation results of the six detection algorithms when integrated into the “Detection Engine” module of the RDE component of the AIDS.

As mentioned in Section 3.1, the six detection algorithms are integrated into the “detection engine” module of the RDE component. The runtime evaluation results of the six detection algorithms, when measured during various evaluation sessions, are presented in Table 6 and Figure 5.

The runtime evaluation results indicate that among the six algorithms tested, LOF, G_KDE, and MCD display low performance. The inferior performance of these algorithms suggests that these algorithms have been overfitted on the training dataset. On the other hand, OCSVM, PW_KDE, and B_GMM demonstrate relatively good performance, effectively distinguishing between attack incidents and non-attack incidents. Even though the evaluation metrics of these three algorithms indicate that overfitting may have occurred, they still exhibit adequate performance overall.

### 3.4. Runtime CPU and Memory Usage Measurements

**Figure 6:**
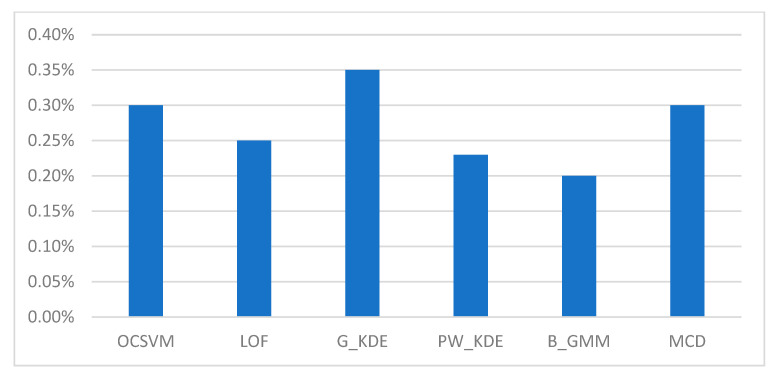
CPU consumption of the RDE component for different detection algorithms.

**Figure 7:**
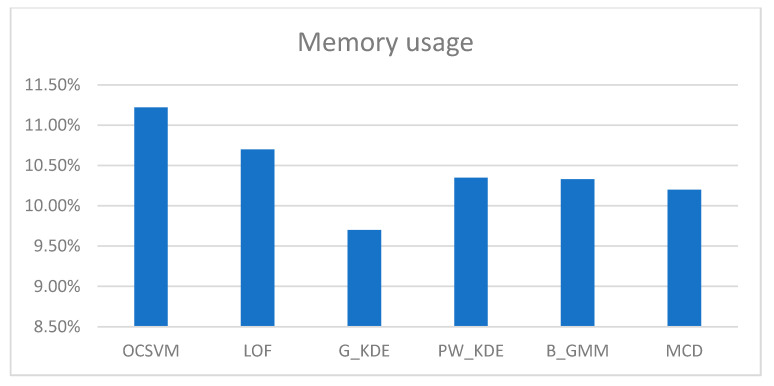
Memory consumption of the RDE component for different detection algorithms.

We executed the “cpu-mem usage” script in two scenarios: (a) on the gateway while the RDE component was running, and on (b) the IoMT device while the MDA component was running. The CPU and memory consumption of the RDE component, when running on the gateway, for different detection algorithms during runtime are shown in Table 7 and depicted in Figure 6 and Figure 7.

In addition, the CPU and memory usage of the MDA component on the IoMT device hosting it were approximately 0.01% and 0.05%, respectively. The measurements taken during the runtime operation of the AIDS indicate that the implemented RDE component requires a minimal percentage (i.e., less than 1%) of the available CPU resources and a moderate percentage of the available memory. Moreover, it is clear that the computational cost of the MDA component, designed for resource-constrained IoMT devices, is further minimized (i.e., less than 0.1%). In addition, as shown in Table 7, the resource requirements during runtime for the RDE component are not significantly affected by the type of algorithm used in the “detection engine” module. Therefore, the implemented AIDS represents a lightweight solution for detecting intrusions targeting resource-constrained IoMT devices.

## 4. Comparison with Existing Works

As mentioned in the introduction, currently, as far as we are aware, only a limited number of AIDSs (i.e., [10,11,12,13,14,15,16]) exist in the literature for protecting resource-limited IoMT devices within IoMT networks. In addition, only two of them (i.e., [13,14]) appear to have been implemented. However, they have not been evaluated during runtime. In this context, our work is compared only to the works in [13,14].

Firstly, in [13], a simulated topology is used to generate datasets to train only one OCSVM model for identifying cyberattacks. The performance of the trained OCSVM model was measured using the recall metric, which ranged from 60.8% to 93.4% for different types of attacks (i.e., rank attacks, version number modification attacks, and flooding attacks). In comparison, in our work, as mentioned in Section 3.3, the three best-performing models (i.e., OCSVM, PW_KDE, and B_GMM) showed better performances, with values ranging from 80% to 97% for the corresponding recall metric (i.e., recall on abnormal class) for different types of attacks (i.e., DoS attacks, CPU exhaustion attacks, and memory exhaustion attacks).

On the other hand, in [14], a smart healthcare testbed is used to generate two datasets with the purpose of training several supervised and unsupervised ML algorithms for identifying cyberattacks (i.e., man-in-the-middle attacks and DoS attacks). This work and the work in [14] are comparable only in the detection of DoS attacks. Table 8 compares (a) the performance evaluation metrics for the best supervised model (i.e., random forest—RF) from the work in [14] and (b) the runtime evaluation metrics for the best novelty detection model (i.e., OCSVM) mentioned in Section 3.4 of this manuscript.

Table 8 shows that the RF model from the work in [14] has a better performance than the novelty detection model of this work. This is an expected outcome as conventional supervised ML algorithms, such as RF, focus more on the classification of a new record into one of the classes for which the classification algorithms have been trained [17]. Conventional classification ML algorithms are capable of accurate predictions in cases where a new record is included in the training dataset. In contrast, conventional classification ML algorithms cannot make accurate predictions if a new record is not part of the training dataset.

On the other hand, novelty detection and outlier detection algorithms are trained mostly on records from a single known class. This, in turn, means that novelty detection and outlier detection algorithms can assess whether a new record is similar to the training records or not [17]. In essence, the main advantage of using novelty detection and outlier detection algorithms in this work is their ability to detect both known and unknown attacks (i.e., unknown to the training data).

This work advances the state-of-the-art in two ways. Firstly, our proposed AIDS is evaluated during runtime in contrast to the state-of-the-art AIDSs that do not include a runtime performance evaluation. Secondly, we use novelty detection and outlier detection algorithms for detecting both known and unknown (i.e., unknown to the training data) attack patterns. In parallel, the computational costs of the implemented AIDS are kept at low levels, meaning that the proposed AIDS constitutes a lightweight security solution that is suitable for IoMT networks and their resource-constrained IoMT devices.

## 5. Conclusions

In this paper, an AIDS specifically designed for resource-constrained devices within IoMT networks was proposed. The design of the proposed lightweight AIDS was presented along with implementation details. The proposed AIDS leverages novelty detection and outlier detection algorithms instead of conventional classification algorithms. The runtime performance of the proposed AIDS was evaluated using an IoMT testbed and dataset developed in our previous works [18,19]. The runtime evaluation results showed that the implemented AIDS can achieve (a) enhanced detection performance against both known and unknown attack patterns and (b) minimal computational costs, with CPU and memory usage for the MDA component remaining below 0.1%.

For future work, we aim to investigate additional outlier detection algorithms that are available in the PyOD [35] library for intrusion detection purposes. In addition, we plan to investigate (a) various combinations (i.e., ensembles) of novelty detection algorithms and/or outlier detection algorithms, (b) deep learning (DL) algorithms, and (c) the possibility of integrating novelty/outlier detection algorithms with conventional classification ML algorithms in an effort to improve the intrusion detection capabilities of the proposed AIDS while keeping the computational cost at low levels as it would need to run on a resource-constrained gateway in an IoMT network (e.g., Raspberry Pi 4 device). The newly explored detection algorithms could be integrated into the detection engine of the proposed AIDS, allowing us to evaluate their performance in detecting intrusions and their computational overhead during runtime.

Furthermore, we aim to experiment with various feature importance and feature reduction methods in order to discover the important features among the full set of features used in the training and decision-making processes. Through these experiments, we will be able to pinpoint combinations of features that can be used in the training and decision-making processes to enhance the performance of the proposed AIDS in detecting intrusions during runtime.

Moreover, we intend to extend the implementation of the MDA component so that it can operate on IoMT devices without requiring a Linux-based OS. For example, popular IoT OSes such as Contiki OS [38] and Zephyr RTOS [39] could be supported. In parallel, the IoMT testbed from our previous work [18] needs to be extended to include devices supporting a wide range of OSes, such as integrating Tmote Sky devices [40] running the Contiki OS.

## Figures and Tables

**Table 1:** Summary of features collected by the “Data Collection” module of the MDA component.

Feature Name	Index	Description
timestamp	1	Time in milliseconds when the current record was collected.
user_ticks	2	Duration in ticks ^1^ that the CPU has been in user mode after system boot.
nice_ticks	3	Duration in ticks ^1^ that the CPU has been in user mode with low priority after system boot.
system_ticks	4	Duration in ticks ^1^ that the CPU has been in system mode after system boot.
idle_ticks	5	Duration in ticks ^1^ that the CPU has been idling after system boot.
iowait_ticks	6	Duration in ticks ^1^ that the CPU has been waiting for I/O to complete after system boot.
irq_ticks	7	Duration in ticks ^1^ that the CPU has been servicing interrupts after system boot.
softirq_ticks	8	Duration in ticks ^1^ that the CPU has been servicing software interrupts after system boot.
steal_ticks	9	Duration in ticks ^1^ that the CPU has been spending in other operating systems when running in a virtualized environment after system boot.
guest_ticks	10	Duration in ticks ^1^ that the CPU has been running a virtual CPU for guest operating systems under the control of the Linux kernel after system boot.
guest_nice_ticks	11	Duration in ticks ^1^ that the CPU has been running a niced (low priority) virtual CPU for guest operating systems under the control of the Linux kernel after system boot.
intr	12	Number of interrupts serviced after system boot.
ctxt	13	Number of context switches that the system has undergone after system boot.
processes	14	Number of newly created processes after system boot.
procs_running	15	Number of processes in a runnable state.
softirq	16	Number of software interrupts serviced after system boot.
mem_total	17	Size of total usable RAM.
mem_free	18	Sum of sizes of free memory in the low-memory region and the high-memory region.
mem_available	19	Estimated size of memory available for starting new applications without swapping.
mem_cached	20	Size of memory used for caching files read from the disk.
mem_active	21	Size of memory that is used frequently and usually not reclaimed unless absolutely necessary.
mem_inactive	22	Size of memory that is used less frequently and can be reclaimed for other purposes.
mem_slab	23	Size of memory that is used as a cache for in-kernel data structures.
mem_kernel_stack	24	Size of memory allocated to kernel stacks.
mem_pagetables	25	Size of memory dedicated to the lowest level of page tables.
reads	26	Number of total read operations that have been completed successfully after system boot.
sectors_rd_num	27	Number of total sectors that have been read successfully after system boot.
msecs_rd	28	Number of milliseconds spent during read operations after system boot.
writes	29	Number of total write operations that have been completed successfully after system boot.
sectors_wr_num	30	Number of total sectors that have been written successfully after system boot.
msecs_wr	31	Number of milliseconds spent during write operations after system boot.
cur_IOs	32	Number of I/O operations currently in progress.
msecs_io	33	Number of milliseconds spent during I/O operations after system boot.

^1^ Ticks = amount of time measured in units of USER_HZ (1/100ths of a second on most processor architectures). The number of ticks starts from zero during system boot.

**Table 2:** Employed external libraries and short descriptions of their use.

Library	Short Description
JSON-java [27]	This library is used to load JSON objects from text, create JSON objects, and transform JSON objects to text.
Apache HttpComponents (Core & Client) [28]	These libraries were used to send intrusion alerts to a remote host through the HTTP protocol.
Eclipse Paho Java Client [22]	This library is used to create MQTT clients so that (a) intrusion alerts can be sent to a remote host through MQTT and (b) IoMT device behavior data can be received by the connected IoMT device through MQTT.
Apache Commons IOUtils [29]	This library is used to simplify input/output operations when using standard Java classes.
Argparse4j [30]	This library is used to be able to include a command-line argument parser in the implementation of the RDE component.

**Table 3:** Output feature values after the first preprocessing stage.

Feature Name	Index	Description
timestamp_delta	1	Elapsed time in milliseconds between the currently collected record and the previously collected record.
user_ticks_delta	2	Duration in ticks ^1^ that the CPU has been in user mode since the previously collected record.
nice_ticks_delta	3	Duration in ticks ^1^ that the CPU has been in user mode with low priority since the previously collected record.
system_ticks_delta	4	Duration in ticks ^1^ that the CPU has been in system mode since the previously collected record.
idle_ticks_delta	5	Duration in ticks ^1^ that the CPU has been idling since the previously collected record.
iowait_ticks_delta	6	Duration in ticks ^1^ that the CPU has been waiting for I/O to complete since the previously collected record.
irq_ticks_delta	7	Duration in ticks ^1^ that the CPU has been servicing interrupts since the previously collected record.
softirq_ticks_delta	8	Duration in ticks ^1^ that the CPU has been servicing software interrupts since the previously collected record.
steal_ticks_delta	9	Duration in ticks ^1^ that the CPU has been spending in other operating systems when running in a virtualized environment since the previously collected record.
guest_ticks_delta	10	Duration in ticks ^1^ that the CPU has been running a virtual CPU for guest operating systems under the control of the Linux kernel since the previously collected record.
guest_nice_ticks_delta	11	Duration in ticks ^1^ that the CPU has been running a niced (low priority) virtual CPU for guest operating systems under the control of the Linux kernel since the previously collected record.
intr_delta	12	Number of interrupts serviced since the previously collected record.
ctxt_delta	13	Number of context switches that the system has undergone since the previously collected record.
processes_delta	14	Number of newly created processes since the previously collected record.
procs_running	15	Number of processes in a runnable state.
softirq_delta	16	Number of software interrupts serviced since the previously collected record.
mem_total	17	Size of total usable RAM.
mem_free	18	Sum of sizes of free memory in the low-memory region and the high-memory region.
mem_available	19	Estimated size of memory available for starting new applications without swapping.
mem_cached	20	Size of memory used for caching files read from the disk.
mem_active	21	Size of memory that is used frequently and usually not reclaimed unless absolutely necessary.
mem_inactive	22	Size of memory that is used less frequently and can be reclaimed for other purposes.
mem_slab	23	Size of memory that is used as a cache for in-kernel data structures.
mem_kernel_stack	24	Size of memory allocated to kernel stacks.
mem_pagetables	25	Size of memory dedicated to the lowest level of page tables.
reads_delta	26	Number of total read operations that have been completed successfully since the previously collected record.
sectors_rd_num_delta	27	Number of total sectors that have been read successfully since the previously collected record.
msecs_rd_delta	28	Number of milliseconds spent during read operations since the previously collected record.
writes_delta	29	Number of total write operations that have been completed successfully since the previously collected record.
sectors_wr_num_delta	30	Number of total sectors that have been written successfully since the previously collected record.
msecs_wr_delta	31	Number of milliseconds spent during write operations since the previously collected record.
cur_IOs	32	Number of I/O operations currently in progress.
msecs_io_delta	33	Number of milliseconds spent during I/O operations since the previously collected record.

^1^ Ticks = amount of time measured in units of USER_HZ (1/100ths of a second on most processor architectures). The number of ticks starts from zero during system boot.

**Table 4:** Output feature values after the second preprocessing stage of the “Data Preprocessing” module.

Feature Name	Index	Description
user_ticks_perc	1	Percentage of ticks that the CPU has been in user mode during the last behavior sampling period.
nice_ticks_perc	2	Percentage of ticks that the CPU has been in user mode with low priority during the last behavior sampling period.
system_ticks_perc	3	Percentage of ticks that the CPU has been in system mode during the last behavior sampling period.
idle_ticks_perc	4	Percentage of ticks that the CPU has been idling during the last behavior sampling period.
iowait_ticks_perc	5	Percentage of ticks that the CPU has been waiting for I/O to complete during the last behavior sampling period.
irq_ticks_perc	6	Percentage of ticks that the CPU has been servicing interrupts during the last behavior sampling period.
softirq_ticks_perc	7	Percentage of ticks that the CPU has been servicing software interrupts during the last behavior sampling period.
steal_ticks_perc	8	Percentage of ticks that the CPU has been spending in other operating systems when running in a virtualized environment during the last behavior sampling period.
guest_ticks_perc	9	Percentage of ticks that the CPU has been running a virtual CPU for guest operating systems under the control of the Linux kernel during the last behavior sampling period.
guest_nice_ticks_perc	10	Percentage of ticks that the CPU has been running a niced (low priority) virtual CPU for guest operating systems under the control of the Linux kernel during the last behavior sampling period.
intr_per_ms	11	Number of interrupts serviced per millisecond during the last behavior sampling period.
ctxt_per_ms	12	Number of context switches per millisecond that the system has undergone during the last behavior sampling period.
processes_per_ms	13	Number of newly created processes per millisecond during the last behavior sampling period.
procs_running	14	Number of processes in a runnable state.
softirq_per_ms	15	Number of software interrupts serviced per millisecond during the last behavior sampling period.
mem_free_perc	16	Sum of sizes of free memory in the low-memory region and the high-memory region as a percentage of total RAM memory.
mem_available_perc	17	Estimated size of memory available for starting new applications without swapping as a percentage of total RAM memory.
mem_cached_perc	18	Size of memory used for caching files read from the disk as a percentage of total RAM memory.
mem_active_perc	19	Size of memory that is used frequently and usually not reclaimed unless absolutely necessary as a percentage of total RAM memory.
mem_inactive_perc	20	Size of memory that is used less frequently and can be reclaimed for other purposes as a percentage of total RAM memory.
mem_slab_perc	21	Size of memory that is used as a cache for in-kernel data structures as a percentage of total RAM memory.
mem_kernel_stack_perc	22	Size of memory allocated to kernel stacks as a percentage of total RAM memory.
mem_pagetables_perc	23	Size of memory dedicated to the lowest level of page tables as a percentage of total RAM memory.
reads_per_ms	24	Number of total read operations that have been completed successfully per millisecond during the last behavior sampling period.
sectors_rd_num_per_ms	25	Number of total sectors that have been read successfully per millisecond during the last behavior sampling period.
msecs_rd_perc	26	Percentage of milliseconds spent during read operations during the last behavior sampling period.
writes_per_ms	27	Number of total write operations that have been completed successfully per millisecond during the last behavior sampling period.
sectors_wr_num_per_ms	28	Number of total sectors that have been written successfully per millisecond during the last behavior sampling period.
msecs_wr_perc	29	Percentage of milliseconds spent during write operations during the last behavior sampling period.
cur_IOs	30	Number of I/O operations currently in progress.
msecs_io_perc	31	Percentage of milliseconds spent during I/O operations during the last behavior sampling period.

**Table 5:** Hyperparameters used when training the six detection algorithms based on the LDE dataset.

	Algorithm	Hyperparameters
Novelty Detection	OCSVM	nu = 0.01, gamma = 0.02, kernel = rbf
	LOF	algorithm = ball_tree, contamination = auto, metric = euclidean, neighbors = 10, novelty = True
	G_KDE	bandwidth = 0.2, kernel = gaussian, metric = manhattan
	PW_KDE	bandwidth = 0.6, kernel = tophat, metric = euclidean
	B_GMM	components = 2, covariance = full
Outlier Detection	MCD	contamination = 0.1, assume_centered = true

**Table 6:** Runtime evaluation results of the six detection algorithms when integrated into the “Detection Engine” module of the RDE component of the AIDS.

	Algorithm	Area Under ROC Curve	Accuracy	Precision on Normal Class	Recall on Normal Class	F1-Score on Normal Class	Precision on Abnormal Class	Recall on Abnormal Class	F1-Score on Abnormal Class
Novelty Detection	OCSVM	81.85	77.76	97.94	66.06	78.9	62.86	97.64	76.48
	LOF	47.85	36.28	38.3	2.51	4.72	36.2	93.18	52.14
	G_KDE	50.21	37.52	100	0.42	0.83	37.36	100	54.39
	PW_KDE	78.17	77.67	86.73	76.25	81.15	66.4	80.09	72.61
	B_GMM	73.48	67.37	96.76	49.79	65.75	53.3	97.17	68.84
Outlier Detection	MCD	50.56	38.8	70	3.94	7.47	37.66	97.17	34.28

**Table 7:** CPU and Memory consumption of the RDE component for different detection algorithms.

Algorithm	CPU Usage	Memory Usage
OCSVM	0.30%	11.22%
LOF	0.25%	10.7%
G_KDE	0.35%	9.7%
PW_KDE	0.23%	10.35%
B_GMM	0.20%	10.33%
MCD	0.30%	10.2%

**Table 8:** Comparison of metrics for DoS attacks with work in [14].

Work	Model	AUC	Accuracy	Precision	Recall
[14]	RF	100	99.15	99	98.6
This work	OCSVM	81.85	77.76	62.86	97.64

## Data Availability

Data are contained within the article.
